# Vaccinating against a Novel Pathogen: A Critical Review of COVID-19 Vaccine Effectiveness Evidence

**DOI:** 10.3390/microorganisms12010089

**Published:** 2023-12-31

**Authors:** Bernard Black, David B. Thaw

**Affiliations:** 1 Pritzker School of Law and Kellogg School of Management, Northwestern University, Chicago, IL 60201, USA; 2 School of Computing & Information and School of Law, University of Pittsburgh, Pittsburgh, PA 15260, USA

**Keywords:** COVID-19, SARS-CoV-2, vaccine, vaccine efficacy, vaccine effectiveness, vaccine booster, BNT162b2, mRNA1273, Ad26.COV2.S, ChAdOx1-S, SARS-CoV-2 variants

## Abstract

We study the experience with COVID-19 vaccination of an initially naïve population, which can inform planning for vaccination against the next novel, highly transmissible pathogen. We focus on the first two pandemic years (wild strain through Delta), because after the Omicron wave in early 2022, very few people were still SARS-CoV-2-naïve. Almost all were vaccinated, infected, or often both. We review the evidence on COVID-19 vaccine effectiveness (VE) and waning effectiveness over time and the relative effectiveness of the four principal vaccines used in developed Western countries: BNT162b2 (Pfizer-BioNTech), mRNA1273 (Moderna), Ad26.CoV2.S (Johnson&Johnson), and ChAdOx1-S (AstraZeneca). As a basis for our analysis, we conducted a PRISMA-compliant review of all studies on PubMed through 15 August 2022, reporting VE against four endpoints for these four vaccines: any infection, symptomatic infection, hospitalization, and death. The mRNA vaccines (BNT162b2, mRNA1273) had high initial VE against all endpoints but protection waned after approximately six months, with BNT162b2 declining faster than mRNA1273. Both mRNA vaccines outperformed the viral vector vaccines (Ad26.CoV2.S and ChAdOx1-S). A third “booster” dose, roughly six months after the initial doses, substantially reduced symptomatic infection, hospitalization, and death. In hindsight, a third dose should be seen as part of the normal vaccination schedule. Our analysis highlights the importance of the real-time population-level surveillance needed to assess evidence for waning, and the need for rapid regulatory response to this evidence.

## 1. Introduction

The COVID-19 pandemic was the first global pandemic in the era of modern air travel, involving a novel, highly transmissible respiratory pathogen, with significant rates of severe disease and death. Vaccines, developed in record time, became a crucial means of preventing severe disease. Masking, lockdowns, remote work, and other mitigation measures slowed the spread of infections and reduced mortality rates, but in the end, these measures were only stopgaps. While the origins of SARS-CoV-2 remain debated, the risk from future respiratory pathogens remains high. A future pathogen could have some or all of the features that made COVID-19 so difficult to control, including asymptomatic spread, aerosol spread, high transmissibility, and rapid evolution to evade immune response even when the immune system has been primed by vaccination or prior infection. 

Thus, it is critical to use the experience with COVID-19 to study vaccine effectiveness and the timing of vaccine doses, as a guide to planning for the next major respiratory pathogen. With that goal in mind, we review the COVID-19 vaccination campaign, including both the initial, primary vaccination and a booster dose. The extensive research on the COVID-19 vaccines provides a unique opportunity to study vaccination practices against a novel respiratory pathogen, including vaccine dosage and timing, vaccine waning over time, and how to measure vaccine effectiveness (VE), including against which endpoints. 

Here, we provide an installment on the broader project of learning lessons from this pandemic, to guide planning for the next one. We focus on what is known about initial and longer-term VE for four leading COVID-19 vaccines against four different endpoints (infection, symptomatic infection, hospitalization, and death). We study and compare the four COVID-19 vaccines that were widely used in the United States (US), Canada, Israel, the United Kingdom (UK), and the European Union. Two were mRNA-based: BNT162b2 (Pfizer-BioNTech) and mRNA1273 (Moderna). Two used an adenovirus vector: Ad26.COV2.S (Johnson&Johnson, below, J&J) and ChAdOx1-S (AstraZeneca). BNT162b2, mRNA1273, and ChAdOx1-S are two-dose vaccines; Ad26.CoV2.S is single-dose.

The first U.S. vaccine authorizations were on 11 December 2020, for BNT162b2 and a week later for mRNA1273 [1,2]. Both vaccines were widely used in many countries. Ad26.CoV2 received U.S. authorization in February 2021 [3] and was used primarily in the U.S. ChAdOx1-S was authorized in the UK on 30 December 2020 [4] and soon thereafter in the European Union. It was not authorized in the U.S. but was widely used in other countries. 

We focus on the period from initial vaccine authorization through the end of 2021, during which SARS-CoV-2 infection came primarily from early virus variants through roughly February 2021, then primarily from the Alpha variant through June 2021, and primarily from the Delta variant for the rest of 2021. We do not study the Omicron-dominant period that began at the very end of 2021, both because after 2021, there is limited information about infection rates due to widespread at-home testing, and because by early 2022, almost the entire population was vaccinated, infected, or both, and thus no longer naïve to the SARS-CoV-2 virus [for U.S. evidence, see [5,6,7]]. 

This review reports vaccine-specific evidence from studies known to us or available on PubMed through 15 August 2022, from both clinical trials and observational studies; including evidence on waning VE following initial vaccination and the value of a third, booster dose against the Delta (VoC B.1.617.2) variant. 

The use and timing of booster doses was initially controversial, partly reflecting disagreement on the goals of vaccination [8]. Booster skeptics asserted that the principal goal of vaccination should be to prevent severe disease and death, and wanted to wait for strong evidence of waning against severe disease before authorizing boosters. Proponents of faster booster rollout were willing to accept imperfect evidence on waning in the middle of an ongoing pandemic. The booster proponents were also more willing to provide boosters to younger people, not at high risk of severe disease, to limit the spread of infection to older, higher risk people (see [9] for evidence supporting this strategy). Neither side could draw on experience with a prior, novel, highly transmissible respiratory pathogen with significant mortality. The most relevant prior pandemic, the 1918 influenza pandemic, predated modern air travel and vaccine development. This review provides such evidence by examining COVID-19 vaccine effectiveness from widespread deployment of vaccines in early 2021 through the emergence of the Omicron variant at the end of 2021.

The main contributions of this review, relative to the principal prior review [10], are that (i) this review covers many more studies over a longer period; (ii) the prior review reported only “minimal” waning against severe disease (thus supporting booster skeptics) and did not study death as an endpoint; and (iii) the prior review did not compare vaccines. This review provides strong evidence of progressive waning of VE against all endpoints, including hospitalization and death. VE for BNT162b2 shows a noticeable decline beginning at 4–5 months after initial vaccination; mRNA1273 wanes somewhat more slowly. ChAdOx1-S has both lower initial VE than the mRNA vaccines and substantial waning; Ad26.COV2.S has much lower initial VE than ChAdOx1-S but less evidence of waning. Our data thus support a ranking of vaccines based on near and medium-term VE hierarchy: mRNA1273 > BNT162b2 VE > ChAdOx1-S > Ad26.COV2.S; and 2 initial doses > 1 dose.

We also report evidence for booster value. As we show below (Table 5), an mRNA booster substantially increases VE for symptomatic infection for all ages and for hospitalization and death for ages 60+. This supports the value of a booster dose, especially for older adults, starting 5–6 months after vaccination. Less definitive evidence suggests booster waning and the value of a fourth dose for ages 60+ (e.g., [11,12]). 

An innovation in this review is to use remaining risk instead of VE (RR is defined as 1 − VE) as a core measure of interest, especially against severe disease [13]. For highly effective vaccines, a small drop in VE can imply a large percentage increase in RR. For example, a drop in VE against death from 95% to 90% implies a doubling in RR from 5% to 10%, and thus a doubling in mortality risk for among vaccinated people. It is easier to observe evidence for waning in the data and understand its significance for severe disease, by studying RR rather than VE. RRs can also provide a basis for comparing vaccines that is more sensitive than VE to moderate differences in remaining risk. We speculate that a focus on VE could explain why both individual studies (e.g., [14,15]) and the principal prior review [10] downplayed the evidence on waning and the differences between vaccines, which this review finds and highlights.

The prior reviews [16,17,18], other than [10], are narrow in scope; none of them address waning. The review in [16] does not address waning; the review in [17] covers only early 2021 studies, before waning had occurred; and the review in [18] covers only randomized trials.

## 2. Materials and Methods

### 2.1. Scope of Review and Literature Search

To be included in this review, a study needed to (i) report vaccine efficacy (for a randomized trial) or VE (for an observational study) for full primary vaccination (two doses for mRNA1273, BNT162b2 VE, and ChAdOx1-S; one dose for Ad26.COV2); (ii) use standard timing between doses (4 weeks for mRNA1273 and ChAdOx1-S; 3 weeks for BNT162b2; not applicable for Ad26.COV2); and (iii) report evidence for one or more of the four endpoints. The requirement for standard timing of initial doses excluded some studies in countries, notably the U.K. and Canada, which in early 2021 spaced out the time between the first and second dose in order to reach more people sooner with a first dose. We accepted studies using a test-negative design, including retrospective studies, despite the known potential bias in this design [19], because the test-negative design is the dominant design used in observational studies. 

The exclusion criteria were (i) the source reported vaccine-specific results; (ii) the source reported time since vaccination with enough granularity so that we could assess evidence for waning; (iii) the sample was large enough to provide reasonable precision for the reported endpoint(s) (we generally required at least 5000 vaccinated people and 5000 controls); (iv) the sample did not have apparent biases that could affect generalization (as would be the case for studies of specific populations such as healthcare workers, military veterans, nursing home residents, children, prisoners, and people who visited the emergency department or who were already hospitalized); and (v) the study included a control group without obvious bias, relative to the treatment group. See Supplementary Materials for additional inclusion and exclusion details.

Since early 2021, the authors manually tracked COVID-19 studies. The studies identified in this way were combined with a PRISMA-compliant review of all papers indexed by PubMed (including preprints) from 1 December 2020 through 15 August 2022, using search criteria which required the title, abstract, or keywords to refer to (1) disease or pathogen name; (2) one or more of the studied vaccines; and (3) vaccine efficacy or vaccine effectiveness. This initial search returned >10,000 papers; it was therefore modified to exclude papers with titles referring to adverse effects, specific populations, high-risk conditions such as obesity, diabetes, and immune-compromised, and antibody levels, which are outside of the scope of this review. Comments, responses, and reviews were excluded as not providing primary evidence. See Supplementary Materials for search details. 

The revised PubMed search returned 1169 results, which were screened based on title and abstract. This screening produced 183 candidates (89 new, 94 previously identified in our manual review), which were retrieved and evaluated against the inclusion and exclusion criteria. Sixty-three papers passed this assessment (27 new, 36 previously identified). Of the 18 studies included in the prior review in [10], 12 are included here; the other 6 did not meet our criteria. The Supplementary Materials include a PRISMA flowchart. 

### 2.2. Data Limitations, Implications for Inclusion, and Presentation of Results

An ideal VE study would (i) report vaccine-specific and ideally variant-specific evidence; (ii) report the time of both full initial vaccination (below, simply “vaccination”) and the relevant endpoint; (iii) include a matched, unvaccinated control group; (iv) cover a population-representative sample, large enough to provide reasonably tight confidence intervals (CIs); (v) report VE or RR for standard, well-defined endpoint(s); (vi) report results within age ranges; and (vii) study a post-vaccination period long enough to allow more severe endpoints to be reached. For example, studying post vaccination mortality only for 30–40 days after vaccination or after a booster, as some studies do (e.g., [11,20]) is insufficient), given the typical lags from vaccination to when the vaccine is fully effective and from infection to death [21]. Even the best available studies do not achieve all of this. Therefore, compromises are needed in assessing which studies to rely on and which questions they can answer. 

The principal analysis addresses the limited granularity of data on the time since vaccination by grouping “early” evidence (up to 120 days since vaccination) and “late” evidence (after 120 days). We do not report results by gender or age range. The included studies are consistent with gender having only modest effects on VE and age having only modest effects on VE against the infection endpoints (e.g., [22,23]). More recent studies provide evidence that waning VE against hospitalization and death occurred principally for ages 60+ [11,24,25]. Most studies report VE for the whole adult population, without controlling for prior infection. When VE was reported with-versus-without prior infection, we used the without-prior-infection data. When multiple protocols were used, the lower VE rate is reported. 

## 3. Results

Our review includes the initial clinical trials, as well as observational studies covering the period through for end of 2021. It includes data from late 2021 on booster VE against the Delta variant. 

### 3.1. Empirical Challenges and Choice of Endpoints

A major challenge in analyzing data across countries, trials, and observational studies is varying definitions of illness severity. These include asymptomatic, symptomatic, mild, requiring medical intervention, moderate, mild to moderate, serious, severe, moderate to severe, hospitalization, admission to an intensive care unit (ICU), critical, and death, among others. Definitions of the same term can vary across nations and studies. Four severity categories emerged from our review of the available studies as the most feasible to examine: (1) any infection (identified through a positive SARS-CoV-2 test); (2) symptomatic infection (infection plus presence of COVID-19 symptoms); (3) hospitalization, defined as symptomatic infection plus inpatient admittance; and (4) death with COVID-19 as a primary cause. For studies which report data for “severe disease” but not hospitalization, we generally assume VE for hospitalization equals reported VE for severe disease. It often required judgment to assess whether reported VE against infection was better understood as being against any infection or against symptomatic infection. 

### 3.2. Evidence from Phase 3 Clinical Trials

The Phase 3 vaccine trials, summarized in Table 1, provide evidence for VE against symptomatic infection against the then-prevalent variants, principally the original “wild” variant and immediate descendants. The primary endpoint for the trials of all four vaccines was symptomatic infection. There were too few hospitalizations and deaths to permit more than a rough assessment of efficacy for these outcomes. The trials were not sized and could not feasibly have been sized to have reasonable power to assess efficacy against these less-common events.

This and later tables report point estimates from the indicated studies. Where there are multiple estimates from a single study, or from multiple studies, we report the range of the point estimates. 

The Phase 3 trial results were highly promising, especially for the mRNA vaccines. Efficacy against symptomatic disease was high, as was the apparent efficacy against hospitalization or death. The viral vector vaccines (Ad26.CoV2.S and ChAdOx1-S) showed lower efficacy against symptomatic disease, but performed strongly against hospitalization or death, especially ChAdOx1-S.

### 3.3. Evidence from Observational Studies on VE Soon within 120 Days after Vaccination

Table 2 summarizes evidence from observational studies of VE in the general population within 120 days after vaccination. The data from Israel on BNT162b2 is particularly compelling, given high-quality, population-level data, and several excellent research groups. Qatar also vaccinated principally with BNT162b2. It has similar data quality but a much younger population (91% under age 50), so may be less representative of outcomes for older adults. Data on ChAdOx1-S are limited because many UK studies did not report vaccine-specific results and the UK used an extended time interval between doses, so some UK studies did not satisfy the inclusion criteria.

The principal relevant variants for these studies were the late-2020, Alpha, and Beta variants.

Overall, the evidence for the mRNA vaccines and ChAdOx1-S was consistent with the clinical trials for any infection and symptomatic infection, although some observational studies reported a lower VE than found in the trials. The early studies confirmed the superior performance for the mRNA vaccines against symptomatic infection, compared to the viral vector vaccines.

The early observational studies also provided large-sample evidence, not available from the initial trials, of strong performance for all four vaccines against hospitalization and death. Anecdotal evidence suggested that most vaccinated persons who required hospitalization were very old or had major comorbidities. The mRNA vaccines were more protective than the viral vector vaccines, and VE for the one-dose Ad26.CoV2.S vaccine against hospitalization and death was below the levels seen for the other vaccines.

### 3.4. Evidence on Waning, Principally against Delta Variant

By July 2021, the COVID-19 situation had greatly changed. The Delta variant had become dominant, and Israeli data provided evidence that VE for BNT162b2 had declined substantially against all outcomes by 5–6 months post-vaccination. Table 3 summarizes the evidence on VE more than 120 days after vaccination. Delta became dominant over the same period in which VE was waning. From the available data, it is not feasible to separate waning into the waning that would have occurred against earlier variants, additional or more rapid waning against Delta, and lower VE against Delta than against earlier variants. The limited available evidence supports waning against both Delta and earlier variants, as well as lower VE against Delta, for both symptomatic infection and hospitalization [22,50,51,52,53].

The 120-day lower bound was chosen based on evidence of clinically important waning beginning around then, our assessment of the time periods since vaccination for which the included studies reported data, and the small number of studies presenting data for a longer period since vaccination or permitting finer decomposition. Waning was progressive, and the Supplementary Materials includes several graphs from individual studies that show this (e.g., [22,51]). Since waning is progressive, the point estimates in Table 3 will overstate VE for periods substantially longer than 120 days after initial vaccination.

Table 3 provides evidence of waning for all vaccines across all outcomes. It also provides further evidence for a comparative ranking of vaccines. mRNA1273 wanes more slowly than BNT162b2, and the mRNA vaccines continue to have higher VE than the viral vector vaccines against hospitalization and death.

### 3.5. Evidence for Important Waning against Severe Disease and Death

In the summer and fall of 2021, booster skeptics argued against the need for boosters, citing studies, including those reported in Table 3, that showed substantial VE against severe disease and death and modest percentage declines from the initial VE percentages shown in Table 2. Indeed, the VE declines against hospitalization and death may appear small. However, Table 1, Table 2 and Table 3 adopt the standard practice of reporting VE as a percent reduction in risk, relative to no vaccination. An alternative view, adopted in Table 4, focuses instead on the remaining risk of an outcome (RR = 100% − VE). As Table 4 shows, RR levels are sharply higher after waning, including against hospitalization and death, for BNT162b2, mRNA1273, and ChAdOx1-S. Ad26.CoV2.S does not wane as strongly, but this is from much higher RR levels soon after vaccination.

Consider the death outcome and the midpoints of the ranges in Table 4. For BNT162b2, RR against death increases from 4.5% to 13.3% (roughly tripling). For mRNA1273, RR against death increases from 2.5% to 9.2% (more than tripling, but from a lower base). For ChAdOx1-S, RR against death increases from 6.5% to 19.7% (tripling, from a higher base). These RR increases have major implications for the mortality of vaccinated persons, and thus for the value of a booster dose. Remaining hospitalization risk also rises sharply for all three vaccines.

In Panel B, the mRNA vaccines have a smaller advantage over the viral vector vaccines and perhaps no remaining advantage against infection or symptomatic infection. However, the mRNA vaccines retain a strong advantage over viral vector vaccines against hospitalization and death. When comparing the viral vector vaccines, Ad26.CoV2.S catches up to ChAdOx1-S against hospitalization and death.

The waning effectiveness of vaccines against hospitalization and death was observed in a real-world setting for BNT162b2 in Israel in mid-2021. Israel was one of the first countries to vaccinate its population, almost exclusively with BNT162b2, and thus one of the first to experience waning. Israel responded with a booster campaign, discussed in the next section.

### 3.6. Evidence on Booster Effectiveness against Delta Variant

Data on booster VE are limited to boosters for the mRNA vaccines and VE against to the Delta variant, which became dominant in mid-2021 before boosters were used. ChAdOx1-S was not often used as a booster during the period we studied, and J&J was rarely used as a booster due to its inferior performance for initial vaccination. The most complete data are for BNT162b2 from Israel, which responded to evidence on waning by launching an aggressive booster vaccination campaign, beginning in July 2021, with eligibility starting 5 months after the initial vaccination [9,62]. Studies in countries with later booster rollouts, including the U.S., cannot separate the effects of the booster dose from the large differences in infectiousness and immune evasion between the Delta and Omicron variants.

Table 5 reports evidence on VE for persons receiving a booster relative to vaccinated but unboosted persons (Panel A), as well as more limited evidence on VE for persons receiving a booster relative to unvaccinated people (Panel B).

The estimates of booster effectiveness from Table 5 suggest that the booster dose was able to substantially restore, against the Delta variant, the high protection against hospitalization and death seen for earlier variants soon after vaccination. However, the booster dose was itself subject to waning VE against infection by the Omicron variant [73]. The extent to which the booster would have waned against the Delta variant is not knowable, due to the short time between when boosters became available and the early 2022 emergence of Omicron as the dominant variant.

## 4. Discussion

### 4.1. Developing Vaccines for a Novel Pathogen

The vaccines against COVID-19 were developed, tested in randomized trials for efficacy against symptomatic infection and for safety, and authorized by regulators in record time. The rapid authorization of several vaccines in late 2020 surely saved millions of lives worldwide. However, the speed with which these vaccines were developed, tested, and authorized involved tradeoffs. The short interval between the first and second doses for the two-dose vaccines was established by the manufacturers with an eye to the minimum spacing needed to establish near-term efficacy, rather than with a view toward optimal dose spacing for longer-lasting protection. Based on experience with other vaccines [74], there was always a likelihood that the initial protection would wane and a booster would be needed, even if the extent and speed of waning was yet to be determined. Moreover, the initial trials were not sized to study efficacy against hospitalization and death, given the low population rates for those endpoints.

The initial speed was wonderful and provides a model to build on. But both vaccine manufacturers and public health authorities fell short in planning for the evaluation of potential waning of VE, and in failing to use RR as a metric for evaluating vaccine performance, especially against severe disease. Observational data to assess waning was potentially available on a massive scale, but in most countries, these data were not systematically collected and analyzed.

This review offers evidence on VE and RR against severe disease and on the waning that was actually experienced, against both infection and severe disease. It can guide the response to a future novel pathogen.

### 4.2. Overall Evidence on Booster Value

There is strong evidence of both (i) vaccine waning beginning 4–5 months after initial vaccination and (ii) significantly higher VE (lower RR) following a booster. Waning began before the mid-2021 emergence of the Delta variant, continued during the Delta-dominant period, and may have been accelerated by the transition from Alpha to Delta as the dominant SARS-CoV-2 strain. After initial caution, e.g., [58], the FDA strongly supports the value of a booster dose, with the FDA commissioner and head of vaccines unit writing in May 2022 that “it is critical that patients and caregivers understand the profound benefit of a booster dose of the mRNA vaccines” [75].

The value of a booster against hospitalization and death is seen primarily for ages 60+ (e.g., [62,64]). But older people account for the vast majority of COVID-19 deaths. The real-world implications of booster doses in reducing mortality are large. A recent study uses Israel as a counterfactual for the U.S. and concludes that if the U.S. had matched Israel’s timing for booster authorization, uptake speed, and eventual uptake rates, this would have saved the lives of 29,000 people aged 55+, versus the actual U.S. booster experience. Conversely, never authorizing a booster dose would have cost an additional 41,000 lives [62]. These amounts are substantial fractions of the 106,000 deaths among vaccinated people during the study period. Giving greater weight to RR as a metric, rather than VE, might have led to faster recognition of waning and booster approval.

### 4.3. Re-Examining Vaccine Dose Timing

The evidence for waning VE suggests that, at least for older people, vaccination for SARS-CoV-2 should include an initial two-dose primary series plus at least one booster, 6 months or so after the first two doses. The vaccination pattern of an initial dose or doses and then a gap before an additional dose, is familiar from recommended schedules for other vaccines [76]. The mRNA vaccines were more effective than the viral vector vaccines, at least in developed countries where the cold-chain requirements for these vaccines could be met. Additional booster doses may be appropriate, at least for ages 60+ [12,20,73], but waning VE for a first booster and thus the potential value of a second or subsequent booster cannot be reliably assessed within the time period of this study.

The interval between primary doses and subsequent (booster) dose(s), and whether that interval depends on vaccine type or patient age, are topics for future research. So is the value of mixing and matching vaccines, either across vaccine types (mRNA versus viral vector or other types, such as the Novavax spike-protein based vaccine [77]) or within types (e.g., mRNA1273 vs. BNT162b2). One UK study finds similar VE against infection for ChAdOx1-S boosted with BNT162b2 versus homologous BNT162b2 vaccination plus a booster dose [78]. A further factor is the risk, illustrated by the emergence of Omicron, of the emergence of more infectious or immune-evasive virus variants. Regulatory decisions on when to allow or recommend additional doses also need to take into account the time needed for population rollout. Thus, even if boosting at 6 months was optimal spacing for individuals, earlier *availability* (perhaps at 5 months as Israel decided) would likely be preferable. The optimal number and timing of vaccine doses might be different for the previously infected, who face a lower risk of severe disease [42,79,80].

### 4.4. Comparing across Vaccines: RR for Hospitalization and Death

For both initial vaccination and a booster dose, RR against hospitalization or death provides a valuable, underused metric for comparing vaccines. Using RR as the metric, a clear preference order emerges for both of these endpoints, both before and after waning: mRNA1273 > BNT162b2 > ChAdOx1-S > Ad26.CoV2.S. The RRs for death, measured at midpoints after waning, are 9.2% for mRNA1273; 13.3% for BNT162b2; 19.7% for ChAdOx1-S; and 23.5% for Ad26.CoV2.S. A similar gradient is seen for hospitalization.

When comparing vaccines, the endpoint matters and so does the time since initial vaccination. Ad26.CoV2.S is initially inferior for all endpoints (Table 2) but wanes more slowly and after 120 days is comparable to ChAdOx1-S for infection (Table 3) and only modestly inferior to ChAdOx1-S against hospitalization and death.

However, differences between vaccines seen for primary vaccination may diminish after a booster. A U.S. study compares BNT162b2 and mRNA1273 and finds a BNT162b2/mRNA1273 odds ratio for death of 2.40 (z = 4.26) after waning for primary vaccination, but only 1.25 (insignificant, z = 0.50) after a booster dose [24]. Whether larger differences between vaccines after a booster dose would emerge over a longer time period is unknown.

### 4.5. Population Implications of Booster Use

Our analysis focused on the benefit of a booster dose for those receiving it. But there can also be benefits to others. One benefit is reduced infection spread. If R_t_ (the time-varying mean number of people infected by each initially infected person) exceeds 1, a single infection can lead to a large number of follow-on infections. Even if R_t_ is modestly below 1, a single infection predicts multiple follow-on infections. For example, for an R_t_ of 0.9, each infection predicts roughly five additional infections (0.9 + 0.9^2^ + 0.9^3^ +…). The young, who generally experience less severe illness, can infect the old, who are more likely to experience severe illness [9]. The vaccinated can infect the unvaccinated. The relevant R_t_ is time-varying and unknown, but greater booster uptake should imply lower transmission. Also, at various times during the pandemic, including early 2022, many hospitals were at or beyond normal capacity, leading to higher mortality for both COVID-19 and other conditions. Reducing infections during peak periods can also reduce demand for treatments that are in short supply.

### 4.6. Value of Harmonized Endpoints

There are no generally accepted, -standardized protocols for clinical endpoints for measuring VE. A lesson from this project is the difficulty of reporting data for harmonized endpoints across studies and countries. We propose that useful categories, which researchers should report where feasible, should include any infection, symptomatic infection, hospitalization, death, and ideally a category between hospitalization and death (perhaps admission to intensive care). Even here, there will be uncertainty—deciding which symptoms count as symptomatic infection, the challenge of measuring asymptomatic infection reliably, and different criteria used in different countries for which patients need hospitalization or intensive care. But these categories are more manageable than categories such as “severe” or “critical” disease, which may be feasible to use in a formal trial or a single country [81] but translate poorly across countries and health systems.

### 4.7. Healthy Vaccinee Bias and Behavioral Differences between the Vaccinated and Unvaccinated

The observational studies we rely on generally cannot address differences between vaccinated and unvaccinated people in behavior or underlying health. For example, test-negative designs, which were the principal designs used in the observational studies that we reviewed, suffer from healthy vaccinee bias [82,83], in which healthier people, who face lower background risk of severe disease, are more likely to become vaccinated or boosted. This bias can be substantial, especially for VE against hospitalization or death [13,24]. The implication is that many studies overestimate VE (underestimate RR). Most studies do not match vaccinated to unvaccinated (beyond age and gender), and those that do often do not control for prior infection. Underestimates of RR need not change the relative ranking of vaccines. However, higher RR for initial vaccination implies a greater value for a booster dose [13].

A separate concern is behavioral differences between the vaccinated and unvaccinated, based on their knowledge of their own status. The negative VE point estimates against infection after waning, found in some studies [36,39], could reflect the vaccinated relaxing their guard against infection. Even careful matching cannot address behavior differences between the vaccinated and unvaccinated. However, another possible explanation for negative point estimates is immune imprinting, a hypothesis still under study in which initial (naïve) exposure (whether to the pathogen or through immunization), and possibly subsequent exposure to the same pathogen/vaccine, causes the body’s immune system to “imprint” the virus variant from initial exposure(s) and to under-react to a newer immune-evasive variant by developing fewer new antibodies that target the new variant, and instead continuing to produce antibodies targeted to the initial exposure(s) [84].

### 4.8. The Need for Data Collection Infrastructure

The COVID-19 pandemic was worldwide as was vaccination. Yet the studies that met our inclusion and exclusion criteria generally fell into several limited groups: (i) the initial, multicountry randomized trials and followup studies, which were not sized to have power to measure VE against hospitalization and death; (ii) studies from Israel, Qatar, and the U.K., which have national public health data infrastructure and strong research teams that could exploit this data; (iii) the U.S., which has fragmented data but good research teams who were able to obtain data from individual health systems or individual states; and (iv) other single-country studies, usually from smaller countries with national data, which often was less effectively exploited. These limits on the available studies affect the generalizability of this review.

The limits on available data also underscore the value of data infrastructure and the need to take steps to improve this infrastructure worldwide before the next pandemic. To respond promptly to emerging evidence—for COVID-19, the evidence on waning that emerged beginning in mid-2021—regulators need access to population-level data in real time, including linkages across datasets (e.g., vaccination, infection, hospitalization, death). They also need the willingness to act on initial evidence, that is less complete or definitive than they might want in a non-crisis situation.

## 5. Conclusions

The mRNA-based COVID-19 vaccines were initially highly effective against all endpoints. The viral vector vaccines were also effective but less so, especially Ad26.CoV2.S (Table 1 and Table 2). However, all vaccines waned substantially against all endpoints, over a limited time period after primary vaccination. This is easier to see using RR as the metric instead of VE (Table 4). Data for booster doses of the mRNA vaccines show substantial reduction in RR, which translates into many lives saved among older people who obtained booster doses. Vaccine development and VE assessment could have, but did not, included explicit assessment of waning, using the observational data generated by the worldwide rollout of primary vaccination.

The experience with COVID-19 suggests that even with two-dose primary vaccination, initial vaccination planning for a novel, highly transmissible respiratory pathogen should reflect the potential value of at least one additional dose and should include population-level surveillance plans sufficient to identify, in close to real time, the timing and need for such a dose. Public health authorities also should prepare the ground for a possible additional dose, and not overpromise with regard to the duration of protection from initial vaccination.

## Figures and Tables

**Table 1:** Vaccine efficacy rates against harmonized endpoints in Phase 3 trials.

	Efficacy vs.			
	Any Infection	Symptomatic Infection	Hospitalization	Death
Vaccine				
BNT162b2 (Pfizer-BioNTech)	NR	95%	100% ^b, c, d^	100% ^b, c^
mRNA1273 (Moderna)	NR	94.5%	100% ^b, c^	100% ^b, c^
Ad26.COV2.S (J&J)	59.7%	66.5%	76.7–83.5% ^a^	100% ^b^
ChAdOx1-S (AstraZeneca)	27.3–64.3% ^e^	70.4–74.0%	94.2–100% ^b, c^	100% ^b, c^

Table sources. NR = not reported or not computable from the reported data. Data for BNT162b2 [26], mRNA1273 [27], and Ad26.CoV2.S [28] are from documents provided by the U.S. Food and Drug Administration (FDA) to Vaccines and Related Biological Products Advisory Committee meetings. Additional data for Ad26.COV2.S are from [29,30], which report lower efficacy against hospitalization than the FDA submission. Data for ChAdOx1-S are from [31,32]. ^a^ The Ad26.CoV2.S protocol did not distinguish between actual hospitalizations and people who came to the emergency department but were not hospitalized. ^b^ Results reported as “100%” indicate no qualifying events in the treatment group, and do not imply that the vaccine would achieve actual efficacy of 100% in a larger population. ^c^ Inferred from no adjudicated cases in the treatment group requiring hospitalization. ^d^ The formal Pfizer submission to the FDA reported no hospitalizations among vaccinated people, but 4 individuals with “severe illness”, of whom 1 was in the vaccine group (not hospitalized). The related academic article [33] reported 6 severe cases between 14 and 112 days after receiving vaccine or placebo, of whom 1 was in the vaccine group. ^e^ Protocols for defining asymptomatic infection varied across the countries in the ChAdOx1-S trial, which limits the reliability of these point estimates.

**Table 2:** Early observational evidence on vaccine effectiveness (pre-Delta).

	Efficacy vs.			
	Any Infection	Symptomatic Infection	Hospitalization ^b^	Death ^b^
Vaccine				
BNT162b2	65.1–93.8% ^a^	86.0–97.7%	87.0–100.0%	91.1–100.0%
mRNA1273	65.1–96.4%	84.9–96.3%	90.6–100.0%	96.0–99.0%
Ad26.CoV2.S	64.0–74.2%	NR	71.0–83.5%	78.0–82.8%
ChAdOx1-S	63.1–67.0%	44.5–74.5%	75.7–95.2%	93.0–94.1%

Table sources: [14,23,34,35,36,37,38,39,40,41,42,43,44,45,46,47,48,49,50]. NR = not reported or not computable from the reported data. The samples in [37,38] overlap. ^a^ The value for [36] is an average of estimates for shorter time periods after second dose. ^b^ Results reported as “100%” indicate no qualifying events in the treatment group and do not imply that the vaccine would achieve actual VE of 100% in a larger population.

**Table 3:** VE against harmonized endpoints at last 120 days after vaccination, principally against Delta variant.

	Efficacy vs.			
	Any Infection	Symptomatic Infection	Hospitalization	Death
Vaccine				
BNT162b2	0.0–54·0% ^a, b^	0.0–70.1% ^a, b^	71.5–90.7%	83.0–90.4%
mRNA1273	0.0–80.0% ^b^	52.1–81.9%	61.0–92.3%	88.0–93.7%
Ad26.CoV2.S	36.0%	37.5–64.3%	65.0–80.0%	73.0–80.0%
ChAdOx1-S	NR	0.0–59.0% ^b^	52.3–77.0%	78.7–82.0%

Table sources: [23,36,39,41,43,44,45,53,54,55,56,57,58,59,60]. NR = not reported or not computable from the reported data. ^a^ For BNT162b2, [36] reports negative point estimates for any infection and symptomatic infection as 0.0. Post-authorization clinical trial results for BNT162b2, not included in Table 3 because they predated the Delta variant, found waning against symptomatic infection [61]. ^b^ Insignificant negative point estimates reported in a few studies are reported in this table as 0%.

**Table 4:** Remaining risk (RR): <120 days (Panel A) vs. >120 days (Panel B) after vaccination.

	Remaining Risk (RR) vs.			
	Any Infection	Symptomatic Infection	Hospitalization ^b^	Death ^b^
Panel A: Evidence within 120 days after Initial Vaccination				
BNT162b2	6.2–34.9%	2.3–14.0%	0.0–13.0%	0.0–8.9%
mRNA1273	6.3–34.9%	3.7–15.2%	0.0–9.4%	1.0–4.0%
Ad26.CoV2.S	25.8–36%	NR	15.6–29.0%	17.2–220%
ChAdOx1-S	33.0–36.9%	25.5–55.5%	4.8–24.3%	5.9–7.0%
Panel B. Evidence Over 120 days after Initial Vaccination				
BNT162b2	46.0–100%	29.9–100%	9.3–28.5%	9.6–17.0%
mRNA1273	20.0–100%	18.1–48.8%	7.7–39.0%	6.3–12.0%
Ad26.CoV2.S	64.0%	35.7–62.5%	20.0–35.0%	20.0–27.0%
ChAdOx1-S	NR	41.1–100%	23.0–47.7%	18.0–21.3%

Panel A reports point estimates for remaining risk (RR) within 120 days after initial vaccination, based on the VE estimates in Table 2. Panel B reports point estimates for RR more than 120 days after initial vaccination, thus allowing for waning, based on the VE estimates in Table 3. RR = 100% − VE. NR = not reported or not computable from the reported data. ^b^ Insignificant negative point estimates reported in a few studies are reported in this table as 0%.

**Table 5:** Booster effectiveness.

	Any Infection	Symptomatic Infection	Hospitalization	Death
Panel A. Risk Reduction (Booster vs. Vaccinated)				
**Vaccine**				
BNT162b2	86–91%	75–95%	70–95%	81–97%
mRNA1273		86–89%	82%	
Panel B. VE vs. Unvaccinated				
BNT162b2		90–93%	88–99%	93–99%
mRNA1273		89%	86%	87%

Table sources: Panel A [63,64,65,66,67,68,69,70]. Panel B [24,66,69,71,72]. Panel A reports point estimates for risk reduction for vaccinated plus boosted people versus people vaccinated without booster, to nearest percent, for endpoints with available data. Panel B reports VE for people with initial vaccination plus booster versus. unvaccinated persons.

## Data Availability

Not applicable because this review does not rely on original data.

## Supplementary Materials

Supplementary materials for this article can be downloaded at: https://www.mdpi.com/article/10.3390/microorganisms12010089/s1, They include textual material, as well as the following tables and figures: PRISMA Flowchart; PRISMA Checklist; PRISMA-S Checklist; Table S2.1. Data Sources Satisfying Inclusion Criteria for Table 2—BNT162b2 (Pfizer); Table S2.2: Data Sources Satisfying Inclusion Criteria for Table 2—mRNA1273 (Moderna); Table S2.3: Data Sources Satisfying Inclusion Criteria for Table 2—Ad26.CoV2.S (J&J); Table S2.4 Data Sources Satisfying Inclusion Criteria for Table 2—ChAdOxS-1 (AstraZeneca); Table S3.1 Data Sources Satisfying Inclusion Criteria for Table 3—BNT162b2 (Pfizer); Table S3.2 Data Sources Satisfying Inclusion Criteria for Table 3—mRNA1273 (Moderna); Table S3.3 Data Sources Satisfying Inclusion Criteria for Table 3—Ad26.CoV2.S (J&J); Table S3.4 Data Sources Satisfying Inclusion Criteria for Table 3—ChAdOx1-S (AstraZeneca).
