# Supplementary material for: Vaccinating against a Novel Pathogen: A Critical Review of COVID-19 Vaccine Effectiveness Evidence
Source: Microorganisms. 2023 Dec 31;12(1):89. doi: 10.3390/microorganisms12010089 (PMC10820171; doi:10.3390/microorganisms12010089)
Supplement: Supplementary file 1 [file microorganisms-12-00089-s001.zip › microorganisms-2765884-supplementary.pdf]

Supplementary Materials for

Vaccinating Against a Novel Pathogen: A Critical Review of COVID-19 Vaccine Effectiveness Evidence

Bernard Black, MS, JD*
Northwestern University, Pritzker School of Law and Kellogg School of Management

David B. Thaw, PhD, JD
University of Pittsburgh, School of Computing & Information and School of Law

December 2023

These Supplementary Materials can be downloaded from SSRN at:
http://ssrn.com/abstract=4659854

The paper can be downloaded from SSRN at:
http://ssrn.com/abstract=4155493

* Corresponding author. bblack@northwestern.edu

Supplementary Materials for

Vaccinating Against a Novel Pathogen: A Critical Review of COVID-19 Vaccine Effectiveness Evidence

Bernard Black and David Thaw

Abstract: These Supplementary Materials contain additional details for Black and Thaw, *Vaccinating Against a Novel Pathogen: A Critical Review of COVID-19 Vaccine Effectiveness Evidence.* They contain additional information on the included studies and information on selected studies that provide evidence of waning but did not fully meet the study inclusion and exclusion criteria. They also contain additional details on the inclusion and exclusion criteria and definitions of outcome measures.

Supplementary Materials for

Vaccinating Against a Novel Pathogen: A Critical Review of COVID-19 Vaccine Effectiveness Evidence

Bernard Black and David Thaw

1. Countries from Which Data is Drawn: Strengths and Limitations

Many of the studies which satisfied our inclusion and exclusion criteria come from Israel, the UK, the US, and Qatar. These are the countries which were most likely to produce research that met the inclusion and exclusion criteria. However, we did identify and include other studies that met the inclusion and exclusion criteria. The randomized trials of the four vaccines, and followup studies based on those trials, were multicountry studies. The review also includes studies from Sweden, South Africa, Brazil, India, Canada, Puerto Rico, Italy, Czechia, and Chile. We comment here briefly on each country.

Israel. Many of the more compelling studies come from Israel. Israel uses Pfizer nearly exclusively. It had an especially rapid initial vaccine distribution, which allows more time to assess vaccine efficacy over time; was well ahead of other countries in conducting a major booster campaign; and it has rich, population data from its four health networks, for a population of around 9.2 million people. All resident Israeli citizens must belong to one of these networks. High-quality research comes from the Israeli Health Ministry, which has population-wide data; from the Clalit health network, which covers around 60% of the Israeli population of around 9.2 million people; the Maccabi clinic, with around 2.5 million covered lives, the Leumit health network, and major hospitals. The Israeli population is less diverse than some other countries.

Qatar. Qatar also primarily uses Pfizer and has good population data, but for a smaller, albeit highly diverse population (2.9 million), with fewer studies. The ones we rely on come from a single research group. While the Qatar data are high quality, as is the research group studying Qatar, the Qatar population is very young, with 91% under age 50 (noted as a limitation in Chemiatelly et al., 2021). This limits the generalizability of the Qatar results.

United Kingdom. The UK used principally AstraZeneca and Pfizer. It has population data from their National Health Service, but many UK studies do not separately study Pfizer versus AstraZeneca. The UK vaccine rollout has other features which limit our ability to draw on UK studies: (i) there was generally an extended time between first and second dose, due to a UK decision in early 2021 to use then-limited supply to provide first doses to more people; (ii) the UK is using Pfizer for booster doses, even for those who initially received AstraZeneca; and (iii) the vaccine rollout was slower than in a number of other countries, including Israel, Qatar, and the US.

United States. The U.S. uses Pfizer, Moderna, and J&J. It lacks population data but has individual health systems with substantial size. The Centers for Disease Control and Prevention (CDC) arranged to receive reports from health systems in a number of states which taken together should be reasonably population representative. However, most reports from this consortium do not distinguish between the two mRNA vaccines, which makes them unusable for our study. Data on J&J is limited because the J&J vaccine because available later than the mRNA vaccines, was widely viewed as inferior to the mRNA vaccines, was infrequently used once there was sufficient vaccine supply to let individuals to choose another vaccine,

and was virtually never used as a booster. A notable limitation on U.S. data is the lack of formalized systems for national vaccination recording and infection reporting, which limits the practicality of population-level observational studies (unlike Israel, Qatar, and the UK) and the nature of the research designs and controls for confounding which the available data will permit.

2. Vaccine Safety Profiles

The known significant adverse effects from each of the four vaccines are rare, mostly short-term, almost never fatal, and are far outweighed by vaccine benefit for all adult age groups. At most the relative incidence of adverse effects might suggest using different vaccines for different groups; for example, one might prefer Pfizer instead of Moderna for young men due to lower myocarditis risk.

All vaccines can produce short-term reactions, including local swelling and soreness at the injection site, fever and fatigue usually for a day or two, and rarely anaphylactic shock (Desai, Desai and Loomis, 2021), which can be addressed by having patients wait for 15-30 minutes after vaccination (to allow for treatment if needed).

For the mRNA vaccines the principal more severe side effects are myocarditis and pericarditis, principally in younger men. However, even for this group, the risk of myocarditis (the more serious of the two side effects is around 1 in 6,000 for the second dose, and the myocarditis usually mild, with only one known fatality. That risk and is far outweighed by the many risks from COVID, including myocarditis (Merovich et al., 2021). The risk of myocarditis from a booster dose is lower than from a second dose; this may be related to the longer time interval between second dose and booster, than between the first and second doses (Buchan et al., 2021).

The viral vector vaccines have small risks of stroke, principally in middle-aged women (Schultz et al., 2021), and Guillain-Barre syndrome. These risks can be addressed by preferring the mRNA vaccines for this group; they reinforce the general efficacy advantage of the mRNA vaccines. In May 2022, the U.S. Food and Drug Administration (FDA) withdrew its prior approval of the J&J vaccine (Ad26.CoV2.S), due to a combination of lower efficacy, faster waning, and an inferior safety profile (US FDA, 2022).

3. Implications of Prior Infection

Most vaccines are administered to uninfected persons, and studied for efficacy for an uninfected population. COVID is unique, because vaccination is occurring concurrent with an active pandemic, with much of the population already infected, often recently so. Especially in a population with many already infected persons (plausible US estimates exceed 50%), the decline in vaccine efficacy for persons who are vaccinated but SARS-CoV-2 naïve could exceed population-level estimates (which do not account for the protective effect of prior infection). Also, vaccine efficacy, optimal vaccine dosing, and dose and timing could differ for the uninfected versus the already infected. This is a fruitful area for further research, which we could not address due to lack of studies attempting this decomposition. At a minimum, it is important for studies of vaccine effectiveness to control for known prior infection.

4. Details on Literature Search

We view our approach – scanning the rapidly developing literature on vaccine and booster efficacy for empirically strong studies, supplemented by a PRISMA-compliant review of all papers posted on PubMed with a defined end date, as providing a realistic balance between speed and completeness.

4.1. PubMed Search Query

Original search query (returned 24,104 results as of June 14, 2022):

> ((covid[tiab]) OR (covid-19[tiab]) OR (sars-cov-2[tiab])) AND (vacci*[tiab]) AND ("2020/12/01"[Date - Create] : "2022/03/31"[Date - Create])

As noted in the text, this query was revised to automatically filter out non-relevant results. The final query used as the basis for text Figure 1 was:

> ((covid[tiab]) OR (covid-19[tiab]) OR (sars-cov-2[tiab])) AND (vacci*[tiab]) AND (bnt162b2[tw] OR bnt-162b2[tw] OR mrna1273[tw] OR mrna-1273[tw] OR chadox1s[tw] OR chadox1[tw] OR chadox1-s[tw] OR ad26cov2s[tw] OR ad26.cov2.s[tw]) AND (effica*[tiab] OR effect*[tiab] OR protect*[tiab] OR prevent*[tiab]) AND ("2020/12/01"[Date - Create] : "2022/03/31"[Date - Create]) NOT (pregnan*[ti]) NOT (tcell*[ti] OR t-cell*[ti]) NOT (advers*[ti] OR allerg*[ti] OR myocard*[ti] OR thromb*[ti] OR meningitis[ti]) NOT (cancer*[ti] OR leukemia[ti] OR chemo*[ti] OR diabet*[ti] OR obes*[ti] OR renal[ti] OR kidney[ti] OR dialysis[ti] OR elder*[ti] OR "older adult*"[ti] OR pulmon*[ti] OR transplant*[ti]) NOT (rhesus*[ti] OR hamster*[ti] OR primate*[ti] OR macaque*[ti] OR mice[ti]) NOT (immunogen*[ti] OR antibod*[ti] OR genomic[ti]) NOT ("phase 1*"[ti]) NOT ("comment on"[ti] OR "response to"[ti])

4.2. PRISMA Flowchart

The chart below follows PRISMA standards, and summarizes the results from our prior knowledge of the VE literature (right-hand side) and our PubMed search (left-hand side).

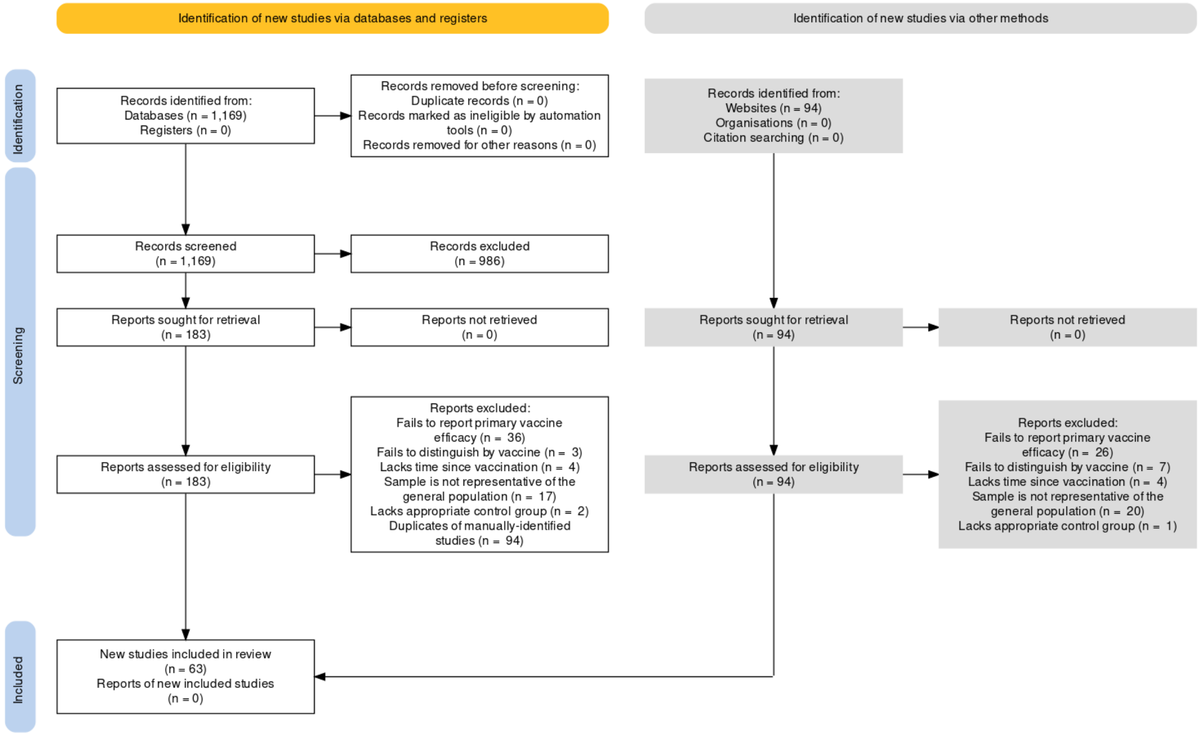

4.3. PRISMA Checklists

The checklists below confirm that our PubMed search complied with PRISMA standards. They are based on Page et. al (2020) and Rethlefsen et. al (2020). For more information, visit: www.prisma-statement.org.

PRISMA Checklist (Page et. al, 2020)

Topic	No.	Item	Location where item is reported
**TITLE**			
**Title**	1	Identify the report as a systematic review.	Title
**ABSTRACT**			
**Abstract**	2	See the PRISMA 2020 for Abstracts checklist	
**INTRODUCTION**			
**Rationale**	3	Describe the rationale for the review in the context of existing knowledge.	1
**Objectives**	4	Provide an explicit statement of the objective(s) or question(s) the review addresses.	2
**METHODS**			
**Eligibility criteria**	5	Specify the inclusion and exclusion criteria for the review and how studies were grouped for the syntheses.	2
**Information sources**	6	Specify all databases, registers, websites, organisations, reference lists and other sources searched or consulted to identify studies. Specify the date when each source was last searched or consulted.	2
**Search strategy**	7	Present the full search strategies for all databases, registers and websites, including any filters and limits used.	2
**Selection process**	8	Specify the methods used to decide whether a study met the inclusion criteria of the review, including how many reviewers screened each record and each report retrieved, whether they worked independently, and if applicable, details of automation tools used in the process.	2
**Data collection process**	9	Specify the methods used to collect data from reports, including how many reviewers collected data from each report, whether they worked independently, any processes for obtaining or confirming data from study investigators, and if applicable, details of automation tools used in the process.	2
**Data items**	10a	List and define all outcomes for which data were sought. Specify whether all results that were compatible with each outcome domain in each study were sought (e.g. for all measures, time points, analyses), and if not, the methods used to decide which results to collect.	3.1
	10b	List and define all other variables for which data were sought (e.g. participant and intervention characteristics, funding sources). Describe any assumptions made about any missing or unclear information.	N/A

Topic	No.	Item	Location where item is reported
**Study risk of bias assessment**	11	Specify the methods used to assess risk of bias in the included studies, including details of the tool(s) used, how many reviewers assessed each study and whether they worked independently, and if applicable, details of automation tools used in the process.	2.2
**Effect measures**	12	Specify for each outcome the effect measure(s) (e.g. risk ratio, mean difference) used in the synthesis or presentation of results.	3
**Synthesis methods**	13a	Describe the processes used to decide which studies were eligible for each synthesis (e.g. tabulating the study intervention characteristics and comparing against the planned groups for each synthesis (item 5)).	2
	13b	Describe any methods required to prepare the data for presentation or synthesis, such as handling of missing summary statistics, or data conversions.	N/A
	13c	Describe any methods used to tabulate or visually display results of individual studies and syntheses.	3.3, 3.4
	13d	Describe any methods used to synthesize results and provide a rationale for the choice(s). If meta-analysis was performed, describe the model(s), method(s) to identify the presence and extent of statistical heterogeneity, and software package(s) used.	N/A
	13e	Describe any methods used to explore possible causes of heterogeneity among study results (e.g. subgroup analysis, meta-regression).	N/A
	13f	Describe any sensitivity analyses conducted to assess robustness of the synthesized results.	N/A
**Reporting bias assessment**	14	Describe any methods used to assess risk of bias due to missing results in a synthesis (arising from reporting biases).	2
**Certainty assessment**	15	Describe any methods used to assess certainty (or confidence) in the body of evidence for an outcome.	2
**RESULTS**			
**Study selection**	16a	Describe the results of the search and selection process, from the number of records identified in the search to the number of studies included in the review, ideally using a flow diagram.	2.1, 2.2, Figure 1
	16b	Cite studies that might appear to meet the inclusion criteria, but which were excluded, and explain why they were excluded.	N/A
**Study characteristics**	17	Cite each included study and present its characteristics.	References
**Risk of bias in studies**	18	Present assessments of risk of bias for each included study.	N/A
**Results of individual studies**	19	For all outcomes, present, for each study: (a) summary statistics for each group (where appropriate) and (b) an effect estimate and its precision (e.g. confidence/credible interval), ideally using structured tables or plots.	Table 2, Table 3
**Results of syntheses**	20a	For each synthesis, briefly summarise the characteristics and risk of bias among contributing studies.	N/A

Topic	No.	Item	Location where item is reported
	20b	Present results of all statistical syntheses conducted. If meta-analysis was done, present for each the summary estimate and its precision (e.g. confidence/credible interval) and measures of statistical heterogeneity. If comparing groups, describe the direction of the effect.	N/A
	20c	Present results of all investigations of possible causes of heterogeneity among study results.	N/A
	20d	Present results of all sensitivity analyses conducted to assess the robustness of the synthesized results.	N/A
**Reporting biases**	21	Present assessments of risk of bias due to missing results (arising from reporting biases) for each synthesis assessed.	2.2
**Certainty of evidence**	22	Present assessments of certainty (or confidence) in the body of evidence for each outcome assessed.	4
**DISCUSSION**			
**Discussion**	23a	Provide a general interpretation of the results in the context of other evidence.	4
	23b	Discuss any limitations of the evidence included in the review.	2.2
	23c	Discuss any limitations of the review processes used.	2.1, 2.2
	23d	Discuss implications of the results for practice, policy, and future research.	4
**OTHER INFORMATION**			
**Registration and protocol**	24a	Provide registration information for the review, including register name and registration number, or state that the review was not registered.	N/A
	24b	Indicate where the review protocol can be accessed, or state that a protocol was not prepared.	N/A
	24c	Describe and explain any amendments to information provided at registration or in the protocol.	N/A
**Support**	25	Describe sources of financial or non-financial support for the review, and the role of the funders or sponsors in the review.	Abstract
**Competing interests**	26	Declare any competing interests of review authors.	Abstract
**Availability of data, code and other materials**	27	Report which of the following are publicly available and where they can be found: template data collection forms; data extracted from included studies; data used for all analyses; analytic code; any other materials used in the review.	N/A

PRISMA-S Checklist (Rethlefsen et. al, 2020)

Section/topic	#	Checklist item	Location(s) Reported
**INFORMATION SOURCES AND METHODS**			
Database name	1	Name each individual database searched, stating the platform for each.	2.1

Section/topic	#	Checklist item	Location(s) Reported
Multi-database searching	2	If databases were searched simultaneously on a single platform, state the name of the platform, listing all of the databases searched.	2.1
Study registries	3	List any study registries searched.	2.1
Online resources and browsing	4	Describe any online or print source purposefully searched or browsed (e.g., tables of contents, print conference proceedings, web sites), and how this was done.	2.1
Citation searching	5	Indicate whether cited references or citing references were examined, and describe any methods used for locating cited/citing references (e.g., browsing reference lists, using a citation index, setting up email alerts for references citing included studies).	2.1
Contacts	6	Indicate whether additional studies or data were sought by contacting authors, experts, manufacturers, or others.	2.1
Other methods	7	Describe any additional information sources or search methods used.	2.1
**SEARCH STRATEGIES**			
Full search strategies	8	Include the search strategies for each database and information source, copied and pasted exactly as run.	2.1, App. 4.1
Limits and restrictions	9	Specify that no limits were used, or describe any limits or restrictions applied to a search (e.g., date or time period, language, study design) and provide justification for their use.	2.1, App. 4.1
Search filters	10	Indicate whether published search filters were used (as originally designed or modified), and if so, cite the filter(s) used.	2.1, App. 4.1
Prior work	11	Indicate when search strategies from other literature reviews were adapted or reused for a substantive part or all of the search, citing the previous review(s).	N/A
Updates	12	Report the methods used to update the search(es) (e.g., rerunning searches, email alerts).	N/A
Dates of searches	13	For each search strategy, provide the date when the last search occurred.	2.1, App. 4.1
**PEER REVIEW**			
Peer review	14	Describe any search peer review process.	N/A
**MANAGING RECORDS**			
Total Records	15	Document the total number of records identified from each database and other information sources.	2.1, Fig. 1, App. 4.1
Deduplication	16	Describe the processes and any software used to deduplicate records from multiple database searches and other information sources.	2.1

4.4. Details for Sources Identified Separately from PubMed

We provide here some additional details on inclusion decisions for our informal scan of the literature.

Studies with limited age range. We considered studies that met the other inclusion criteria but had a limited age range, for the any infection and symptomatic infection endpoints, but not for the hospitalization or death endpoints, because of the strong age gradient for these endpoints.

Smaller studies. We considered studies that included at least 5,000 persons who received a particular vaccine and at least 5,000 unvaccinated controls. We relaxed this limit when no other study was available that addressed the same outcome for the same vaccine.

Studies not written in English. We did not review studies not written in English.

Ongoing clinical trials. The clinical vaccine trials did not stop when data was presented to regulators, leading to some differences in results reported to the FDA and those published later, and to some differences between earlier- and later-published studies.

Astra-Zeneca clinical trials. We rely on the initial pooled analysis of several separate randomized trials of the AstraZeneca vaccine in different countries by Voysey et al. (2021a), rather than their later study which assesses efficacy based on varying the time between the first and second dose (Voysey, 2021b). We rely on the Voysey et al. (2021a) pooled analysis rather than the individual studies they draw from, which include Folegatti et. al (2020), and Ramasamy et. al (2021).

Moderna clinical trial. We rely on the analysis presented to the FDA, rather than the subsequent published study (Baden et al., 2021).

Pfizer clinical trial. We rely both on the analysis presented to the FDA plus, for severe illness, the subsequent published study (Polack et al., 2021).

Studies of booster VE need to be limited to the immune-competent. A study of booster VE must study *booster* doses, not simply third doses. For the immune-compromised, a third dose is considered part of primary vaccination. Including the immune-compromised in a study of three dose VE will produce a downward biased estimate of VE for a booster dose for the immune-competent. Depending on when the study was conducted, the sample of three-dose recipients could be dominated by the immune-compromised, leading to severe downward bias in three-dose VE estimates. We excluded studies of booster doses that did not exclude the immune-compromised and, based on the study period relative to when boosters were authorized, were likely to include a substantial proportion of immune-compromised persons.

5. Need for Vaccine-specific Evidence

A number of studies did not provide vaccine-specific evidence, and thus could not be included in our review. This was an issue particularly for the UK, which used both AstraZeneca and Pfizer extensively, and for CDC studies in the U.S. that did not separately assess Pfizer versus Moderna. The vaccines are different enough, however, to deserve separate analysis. For example, the relative underperformance of AstraZeneca led to a U.K. decision to use Pfizer as a booster, regardless of which vaccine people received initially. The underperformance of J&J, which may partly reflect it being a one-dose vaccine, led to J&J

obtaining U.S. approval for a booster after two months, and to the FDA later withdrawing its approval of J&J.

6. Expanded Results Reporting

This Section provides expanded information regarding the results of this analysis, the criteria used, and the data sources involved.

6.1. Table 1: Vaccine Efficacy Rates Against Harmonized Endpoints in Initial Phase 3 Trials

The Phase 3 trials were primarily conducted in 2020 (reported data is exclusively so for BNT162b2 (Pfizer), mRNA1273 (Moderna), and ChAdOxS-1 (AstraZeneca)). The trials thus had effectively no inclusion of B.1.617.2 (Delta) and at most limited inclusion of B.1.1.7 (Alpha). Furthermore, the BNT162b2 and mRNA1273 trials primarily relied on U.S.-based participants, and efficacy could have been different in other nations which imposed stricter NPIs, had different public health messaging, and may have had different behavioral characteristics among the study population. These factors might account for some of the differences in efficacy seen between the Phase 3 trials (Table 1) and early observational studies (Table 2).

6.2. Table 2: Early Observational Evidence on Vaccine Effectiveness (pre-Delta)

The tables below provide additional detail on the sources used for Table 2. Detailed judgments concerning specific studies, including inclusion decisions and how VE was reported, are indicated in the table with small triangular marks in the upper right corners of some cells, and are available from the authors on request. We excluded Glatstein-Friedman et al (2022) due to the very short post-vaccination time period covered, which could explain their VE percentages, which are far below other studies.

Table S2.1: Data Sources Satisfying Inclusion Criteria for Table 2 – BNT162b2 (Pfizer)

Authors	Country	Journal	Variant(s)	Time Since Vaccination	Any Infection	Symptomatic Infection	Hospitalization	Death
Abu-Raddad, Chemaitelly, et al.	Qatar	NEJM	B.1.1.7	0-30	89.5%		100.0%	100.0%
			B.1.351	0-30	75.0%		100.0%	100.0%
			other	0-30	NR		97.4%	97.4%
Chung, He, Nasreen, et al.	Canada	BMJ		0-99		91.0%		
Andrews, Tessier, et al.	UK	medRxiv	B.1.617.2	7-63		89.8%	98.4%	95.2%
Berec, Smid, Pribylova, et al.	Czech Republi	PLOS One		0-60			90.0%	92.0%
Bernal, Andrews, et al.	UK	NEJM	B.1.1.7	7-120		* 93.7%		
			B.1.617.2	7-120		88.0%		
Chemaitelly, Tang, et al.	Qatar	medRxiv		0-84	65.1%		95.4%	
Dagan, Barda, et al.	Israel	NEJM		7-30	92.0%	94.0%	87.0%	
Glatman-Freedman, Bromberg, Dichtiar, et al.	Israel	EBioMedicine		0-28	92.6%	95.3%	95.4%	93.6%
Haas, Angulo, et al.	Israel	Lancet		0-60	91.5%	97.0%	97.2%	96.7%
				0-53	93.8%	97.7%	98.0%	98.1%
Nordstrom, Ballin, et al.	Sweden	SSRN		0-106 (weighted)		87.1%		
Pawlowski, Lenehan, Puranik, et al.	US	Clinical Advances		0-106		88.0%	88.3%	
Pilishvili, Gierke, et al.	US	NEJM		0-90		88.8%		
Pouwels, Pritchard, Matthews, et al.	UK	Nature Medicine		0-76	82.0%	86.0%		
Robles-Fontan, Neives, Cardona-Gerena, et al	US	Lancet Regional Health - Americas		0-143	87.0%		92.0%	97.0%
Saciuk, Kertes, Mandel, et al.	Israel	Preventive Medicine		0-98	93.0%		93.4%	91.1%
Self, Tenforde, et al.	US	MMWR		14-120			91.0%	
Thompson, Burgess, Naleway, et al.	US	NEJM		0-116	93.0%			
**Summary Statistics**				**Minimum**	65.1%	86.0%	87.0%	91.1%
				**Maximum**	93.8%	97.7%	100.0%	100.0%
				Raw Mean	86.8%	91.2%	94.5%	96.1%
				Raw Median	91.5%	89.8%	95.4%	96.9%
** excluded per protocol of reporting lowest estimate across variants*								

Table S2.2: Data Sources Satisfying Inclusion Criteria for Table 2 – mRNA1273 (Moderna)

Authors	Country	Journal	Variant(s)	Time Since Vaccination	Any Infection	Symptomatic Infection	Hospitalization	Death
Abu-Raddad, Chemaitelly, and Bertollini	Qatar	NEJM		0-150	65.1%	84.8%	91.2%	
Andrews, Tessier, et al.	UK	medRxiv	B.1.617.2	7-63		94.5%	100.0%	
Berec, Smid, Pribylova, et al.	Czech Republic	PLOS One		0-60			94.0%	96.0%
Bruxvoort, Sy, Qian, et al.	US	BMJ	B.1.617.2	14-60	94.1%		97.5%	
			others	14-60	* 98.6%		NR	
Chemaitelly, Yassine, Benslimane, et al.	Qatar	Nature Medicine	*any*	0-120			100.0%	
			B.1.1.7	0-120	* 99.2%			
			B.1.351	0-120	96.4%			
Chung, He, Nasreen, et al.	Canada	BMJ		0-99		94.0%		
Nordstrom, Ballin, et al.	Sweden	SSRN		0-106 (weighted)		88.7%		
Pawlowski, Lenehan, Puranik, et al.	US	Clinical Advances		0-106		92.3%		
Pilishvili, Gierke, et al.	US	NEJM		0-90		96.3%	90.6%	
Robles-Fontan, Neives, Cardona-Gerena, et al	US	Lancet Regional Health - Amer		0-143	90.0%		95.0%	99.0%
Self, Tenforde, et al.	US	MMWR		14-120			93.0%	
Thompson, Burgess, Naleway, et al.	US	NEJM		0-116	82.0%			
**Summary Statistics**				**Minimum**	65.1%	84.8%	90.6%	96.0%
				**Maximum**	96.4%	96.3%	100.0%	99.0%
				Raw Mean	85.5%	91.8%	95.2%	97.5%
				Raw Median	90.0%	93.2%	94.5%	97.5%
** excluded per protocol of reporting lowest estimate across variants*								

Table S2.3: Data Sources Satisfying Inclusion Criteria for Table 2 – Ad26.CoV2.S (J&J)

Authors	Country	Journal	Variant(s)	Time Since Vaccination	Any Infection	Symptomatic Infection	Hospitalization	Death
Berec, Smid, Pribylova, et al.	Czech Republic	PLOS One		0-60			68.0%	68.0%
Corchado-Garcia, Zemmour, Hughes, et al.	US	JAMA NO		0-96	74.2%			
Robles-Fontan, Neives, Cardona-Gerena, et al	US	Lancet Regional Health - Americas		0-143	64.0%		82.0%	78.0%
Sadoff, Gray, Vandebosch, et al. (2021)	*multiple*	NEJM		28			83.5%	
Sadoff, Gray, Vandebosch, et al. (2022)	*multiple*	NEJM		28			74.6%	82.8%
Self, Tenforde, et al.	US	MMWR		0 to less than 120			71.0%	
**Summary Statistics**				**Minimum**	64.0%	0.0%	71.0%	78.0%
				**Maximum**	74.2%	0.0%	83.5%	82.8%
				Raw Mean	69.1%	#DIV/0!	77.8%	80.4%
				Raw Median	69.1%	#NUM!	78.3%	80.4%

Table S2.4: Data Sources Satisfying Inclusion Criteria for Table 2 – ChAdOxS-1 (AstraZeneca)

Authors	Country	Journal	Variant(s)	Time Since Vaccination*	Infection	Symptomatic Infection	Hospitalization	Death
Andrews, Tessier, et al.	UK	medRxiv	B.1.617.2	7-63		66.7%	95.2%	94.1%
Berec, Smid, Pribylova, et al.	Czech Republic	PLOS One		0-60			87.0%	93.0%
Bernal, Andrews, et al.	UK	NEJM	B.1.1.7	7-120		74.5%		
			B.1.617.2	7-120		67.0%		
Katikireddi, Cerqueira-Silva, Vasileiou, et al.	UK (Scotland)			0-111 (weighted)		59.4%	79.5%	
	Brazil			0-111 (weighted)		65.9%	75.7%	
Nordstrom, Ballin, et al.	Sweden	SSRN		0-106 (weighted)		44.5%		
Pouwels, Pritchard, Matthews, et al.	UK	Nature Medicine		0-76	67.0%	70.0%		
Thiruvengadam, Awasthi, Medigeshi, et al.	India	Lancet Inf. Diseases	B.1.617.2	0-116	63.1%		81.5%	
* Reported as number of days since administration of terminal dose of the primary series								
**Summary Statistics**				**Minimum**	63.1%	44.5%	75.7%	93.0%
				**Maximum**	67.0%	74.5%	95.2%	94.1%
				Raw Mean	65.1%	64.0%	83.8%	93.6%
				Raw Median	65.1%	66.7%	81.5%	93.6%

For studies (e.g., Nordstrom, Balin, et al.) that reported results by more limited periods (e.g., one month or less) and did not themselves create weighted averages for "early" and/or "late" VE, the authors performed simple event-weighted averages for those periods consistent with the "early" and "late" periods defined in Tables 2 and 3 of the main text.

6.3. Table 3: Vaccine Efficacy Rates Against Harmonized Endpoints Four-plus Months After Vaccination

Decomposing efficacy decrease between the effect of waning and the effect of B.1.617.2 (Delta) was beyond the scope of our analysis. However as noted in the main text, Keehner et al. (2021) attempted this decomposition using UK data, and found that waning was the more likely cause. In any event, given the dominance of Delta, public health advice would be the same regardless of whether similar waning would have been seen against earlier variants. As discussed in the text, the recent emergence of the Omicron variant appears to increase the value and urgency of a booster dose.

The selection of 120 days (four months) as the dividing line between early and later evidence on efficacy was based on our analysis of the data sources satisfying the inclusion criteria, the time frames used in those studies and an assessment that statistically significant evidence of waning across endpoints begins at around four months. Some studies, notably Chemiatelly et al. (2021), find evidence of substantial waning earlier than 4 months against any infection and symptomatic infection. Using an alternate dividing line, such as the 5 months at which a booster is recommended in Israel or the 6 months at which a booster is recommended in the US, would have reduced the number of usable sources, and would not have affected the overall conclusion on progressive waning of efficacy and the value of a third dose at roughly 5-6 months.

Table S3.1: Data Sources Satisfying Inclusion Criteria for Table 3 – BNT162b2 (Pfizer)

**Authors**	**Country**	**Journal**	**Variant**	**Minimum Time Since Vaccination**	**Any Infection**	**Symptomatic Infection**	**Hospitalization**	**Death**
Andrews	UK	medRxiv		140		69.7%	90.7%	90.4%
Berec, Smid, Pribylova, et al.	Czech Republ	PLOS One		210-240			75.0%	83.0%
Chemaitelly, Tang, et al.	Qatar	medRxiv		175	0.0%	0.0%	71.5%	
Israel Ministry of Health	Israel	VRBPAC Slide Excerpt		180	16.0%	16.0%	82.0%	
Lin, Gu, et al.	US	medRxiv		210		70.1%	87.7%	88.4%
Nordstrom, Ballin, et al.	Sweden	SSRN		107		33.5%		
Robles-Fontan, Neives, Cardona-Gerena, et al	US	Lancet Regional Health - Americas		144	54.0%		81.0%	86.0%
Self, Tenforde, et al.	US	MMWR		120			77.0%	
Tartof, Slezak, et al.	US	Lancet		160	47.0%		88.0%	
**Summary Statistics**				**Minimum**	0.0%	0.0%	71.5%	83.0%
				**Maximum**	54.0%	70.1%	90.7%	90.4%
				Raw Mean	29.3%	37.9%	81.6%	87.0%
				Raw Median	31.5%	33.5%	81.5%	87.2%

Table S3.2: Data Sources Satisfying Inclusion Criteria for Table 3 – mRNA1273 (Moderna)

**Authors**	**Country**	**Journal**	**Variant(s)**	**Time Since Vaccination**	**Any Infection**	**Symptomatic Infection**	**Hospitalization**	**Death**
Abu-Raddad, Chemaitelly, and Bertollini	Qatar	NEJM		150+	-23.0%	51.2%	61.0%	
Berec, Smid, Pribylova, et a	Czech Repu	PLOS One		210-240			81.0%	88.0%
Bruxvoort, Sy, Qian, et al.	US	BMJ	B.1.617.2	151-180	80.0%		NR	
			others	151-180	* 88.7%		NR	
Lin, Gu, et al.	US	medRxiv		210		81.9%	92.3%	93.7%
Nordstrom, Ballin, et al.	Sweden	SSRN		107		67.0%		
Robles-Fontan, Neives, Cardona-Gerena, et al	US	Lancet Regional Health - Americas		144	72.0%		91.0%	93.0%
Self, Tenforde, et al.	US	MMWR		120			92.0%	
**Summary Statistics**				**Minimum**	-23.0%	51.2%	61.0%	88.0%
				**Maximum**	80.0%	81.9%	92.3%	93.7%
				Raw Mean	43.0%	66.7%	83.5%	91.6%
				Raw Median	72.0%	67.0%	91.0%	93.0%
** excluded per protocol of reporting lowest estimate across variants*								

Table S3.3: Data Sources Satisfying Inclusion Criteria for Table 3 – Ad26.CoV2.S (J&J)

Authors	Country	Journal	Variant	Time Since Vaccination	Any Infection	Symptomatic Infection	Hospitalization	Death
Berec, Smid, Pribylova,	Czech Republic	PLOS One		150-180			67.0%	68.0%
Lin, Gu, et al.	US	medRxiv		150		64.3%	80.0%	80.0%
Gray and Becker	South Africa	Sisonke presentation		90 - 120			65.0%	
Robles-Fontan, Neives, Cardona-Gerena, et al	US	Lancet Regional Health - Americas		144	36.0%		67.0%	73.0%
J&J	multi-national	VRBPAC sponsor presentation		150		49.0%	65.0%	
		Unblinded Phase 3 followup		180		37.5%	65.0%	
				210		37.5%	* 85%	
**Summary Statistics**				**Minimum**	36.0%	37.5%	65.0%	73.0%
				**Maximum**	36.0%	64.3%	80.0%	80.0%
				Raw Mean	36.0%	47.1%	68.4%	76.5%
				Raw Median	36.0%	43.3%	65.0%	76.5%
** excluded per protocol of reporting lowest estimate when weighting not possible*								

Table S3.4: Data Sources Satisfying Inclusion Criteria for Table 3 – ChAdOx1-S (AstraZeneca)

Authors	Country	Journal	Variant	Time Since Vaccination*	Any Infection	Symptomatic Infection	Hospitalization	Death
Andrews, Tessier et al.	UK	medRxiv		140		47.3%	77.0%	78.7%
Berec, Smid, Pribylova, et al	Czech Republi	PLOS One		180			70.0%	82.0%
Katikireddi, Cerqueira-Silva,	UK (Scotland)	medRxiv		98-153		47.2%	64.3%	
	Brazil*	medRxiv		98-139		59.0%	52.3%	
Nordstrom, Ballin, et al.	Sweden	SSRN		107		-19.0%		
**Summary Statistics**				**Minimum**	0.0%	-19.0%	52.3%	78.7%
				**Maximum**	0.0%	59.0%	77.0%	82.0%
				Raw Mean	#DIV/0!	33.6%	65.9%	80.4%
				Raw Median	#NUM!	47.2%	67.1%	80.4%
** included in calculation due to treating different country as effectively a separate study*								

For studies (e.g., Nordstrom, Balin, et al.) that reported results by more limited periods (e.g., one month or less) and did not themselves create weighted averages for "early" and/or "late" VE, the authors performed simple event-weighted averages for those periods consistent with the "early" and "late" periods defined in Tables 2 and 3 of the main text.

6.4. Inclusion Criteria

We faced the challenge that some Israeli and U.S. studies report efficacy for severe disease, but not for hospitalization. We made the judgment to report efficacy for these studies under the harmonized hospitalization outcome, based on evidence that: (i) roughly two-thirds of hospitalized Israeli patients are

classified as having severe disease; and (ii) when efficacy is reported for both hospitalization and severe disease, efficacy is very similar for both outcomes (Barda et al., 2021; Bar-On et al., 2021a)). We used a similar approach for other studies that rely on the U.S. National Institutes of Health "critical illness" category.

When hospitalization was not a defined endpoint, and other endpoints were mixed or unclear (e.g., "severe-critical"), such an endpoint was treated as hospitalization if: (1) there were endpoints of lesser severity reported in the study which included inpatient clinical intervention; and (2) there were no endpoints of greater severity reported in the study which both required inpatient clinical intervention and did not require ICU care. Fatalities were included for the "death" endpoint if the study confirmed COVID-19 as a primary cause of death. When symptomatic infection was not a clearly defined endpoint and other endpoints were mixed/unclear, to qualify for the "symptomatic" endpoint the study's similar endpoint had to include: (1) clinically-confirmed COVID-19 illness; (2) diagnosed COVID-19 symptoms; (3) no medical intervention was clinically required (excluding routine analgesic care for patient comfort); and (4) the study had no other endpoints meeting these criteria.

Studies report data based on different start times (e.g., 7 days after terminal dose (the second dose for a two-dose regime, the only dose for J&J), date of terminal dose, 14 days after terminal dose). These are normalized to day "0" defined as 14 days after the terminal dose in the primary series. In some cases, we exercised judgment on how to classify results reported in another way. For example, Chemiatelly et al. (2021) report efficacy of Pfizer vaccination for periods of 0-4, 5-9, 10-14 etc. weeks after second dose. We chose to include the 0-4 week period in reporting efficacy during the first 120 days after full vaccination.

The precision of reported estimates depends on a combination of sample size, the outcome being studied, and COVID prevalence in the population during the study period. In light of these complexities, we did not apply a strict numerical size cutoff, but did exclude a number of U.S. studies that, for example, were limited to a single site, to a convenience sample such as healthcare workers, or both.

6.5. Additional Studies Addressing Waning Efficacy over Time

We discuss here selected additional studies which provide evidence on initial VE, waning over time, or both, but did not meet the inclusion criteria for Tables 2-3.

Bajema et al. (2021) studies vaccine efficacy against hospitalization, for a sample of hospitalized U.S. veterans, primarily male and generally older (median ae 63). We did not include this study because it studies only hospitalization for a predominantly male and older population, which limits generalizability. This study reports somewhat lower initial efficacy against hospitalization during the first 120 days than the studies included in Table 2 (89.6% for Moderna, 86.0 for Pfizer), with mild evidence of waning for periods over 120 days for Moderna (to 86.1%) and stronger waning for Pfizer (to 75.1%).

Cohn et al. (2021) also study U.S. military veterans and provides evidence of waning VE against infection (mostly symptomatic infection, since there was no systematic testing of the study population). The figure below reports hazard ratios from this study, using a Cox proportional hazard model. mRNA1273 efficacy decays more slowly, and Ad26.CoV2.S more rapidly, with BNT162b2 in the middle.

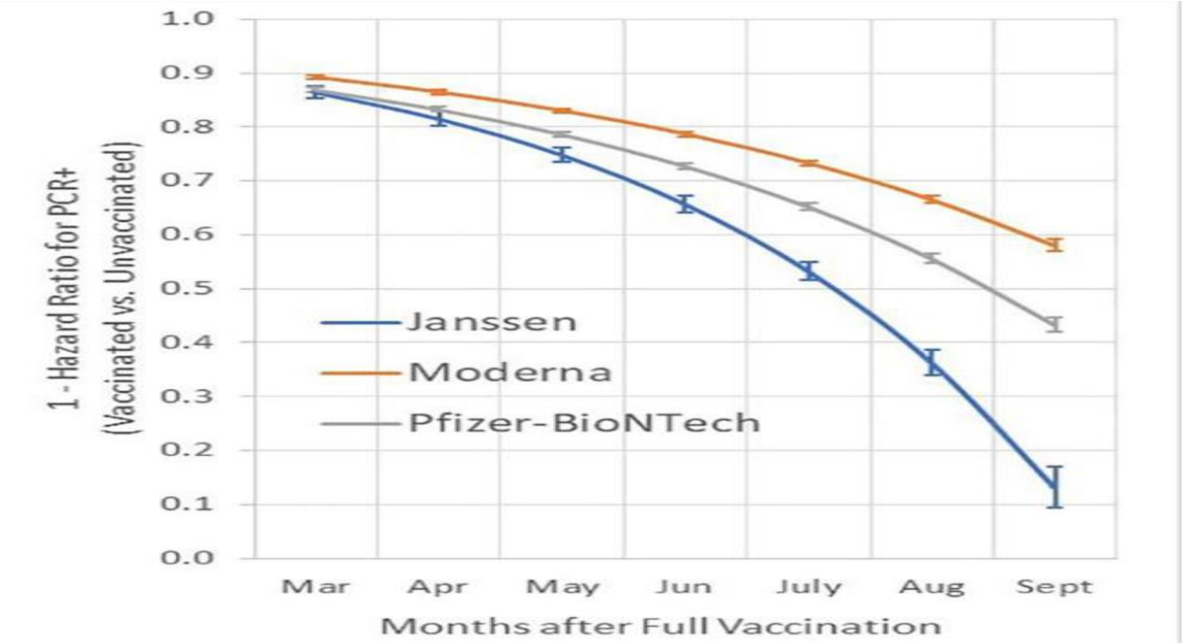

Puranik et al. (2021) provide U.S. evidence from matched vaccinated and unvaccinated cohorts, followed longitudinally in the Mayo Clinic of waning for both Pfizer and Moderna, with stronger waning for Pfizer, over January-July 2021. This study did not satisfy the inclusion criteria because it does not control directly for date of vaccination.

Scobie et al. (2021) report U.S. evidence of waning in 13 states over April-July 2021, but did not meet the inclusion criteria because it does not control for either vaccine type or date of vaccination. Given that the U.S. used Pfizer and Moderna in similar percentages, with much lower (around 4%) use of J&J, this can be understood as effectively a study of the mRNA vaccines.

Thomas et al. (2021) [45] report clinical trial results for Pfizer efficacy through 6 months after vaccination. Because data comes from the Pfizer trial, This study involves an earlier, pre-Delta period than the studies reported in Table 3. Waning protection against symptomatic infection was found, but to a lesser than the Pfizer studies in Table 3; from 96.2% (7 days to 2 months after second dose) to 90.1% (2-4 months after second dose) and 83.7% (4-6 months after second dose). Efficacy against severe disease which we treat as equivalent to hospitalization was reported as 96.7%, (one reported case in vaccine group, breakdown for time since vaccination was not feasible).

Israel et al. (2021) study relative infection rates for matched Pfizer-vaccinated adults in Israel, based on time since vaccination, using data from the Leumit health clinic. They report a steady increase with increasing time since vaccination, summarized in the Figure below. Because this study examines only the vaccinated, it can address potential behavioral differences between vaccinated and unvaccinated persons. This study did not meet the inclusion criteria because it does not compare vaccinated to unvaccinated persons.

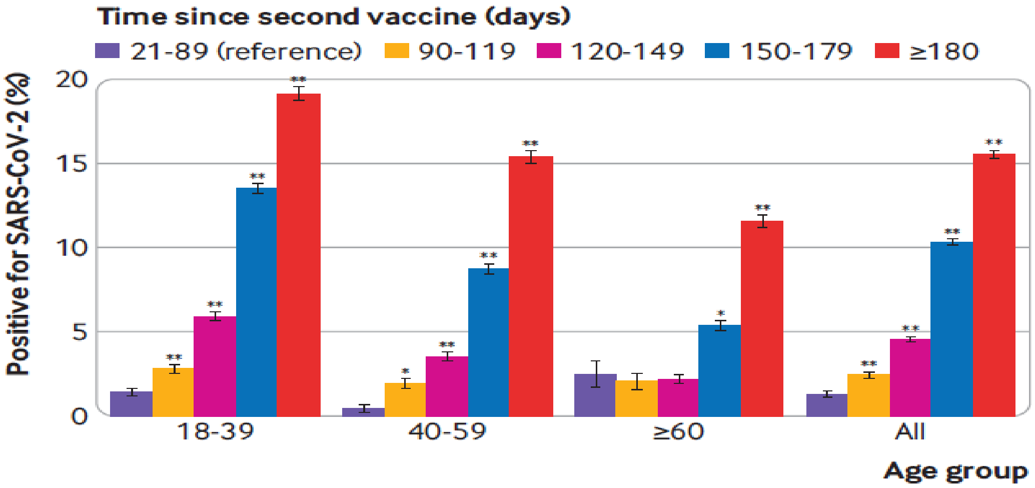

Fig 2 | Comparison of percentage of positive results, according to time elapsed since second vaccine dose, in pre-matched cohort. Error bars=standard error of the proportion. *P<0.01, **P<0.001

Source: Israel et al. (2021), fig. 2.

Mizrahi et al. (2021) usen data from the Maccabi Health Clinic in Israel to study infection rates for persons vaccinated with BNT162b2 early (January-February 2021) versus late (March-April 2021),and report roughly 1.5 times higher infection rates for the early vaccinees.

Goldberg et al. (2021) use population data from the Israeli Health Ministry, and report declining efficacy for BNT162b2 against both any infection and severe disease in July 2021, based on time of initial vaccination. The principal results reported in the text are limited to vaccinated persons, who were vaccinated at different times, and are summarized in the figure below. The authors provide more limited data comparing vaccinated to unvaccinated persons in an Appendix. We excluded this study because the main results do not compare vaccinated to unvaccinated persons.

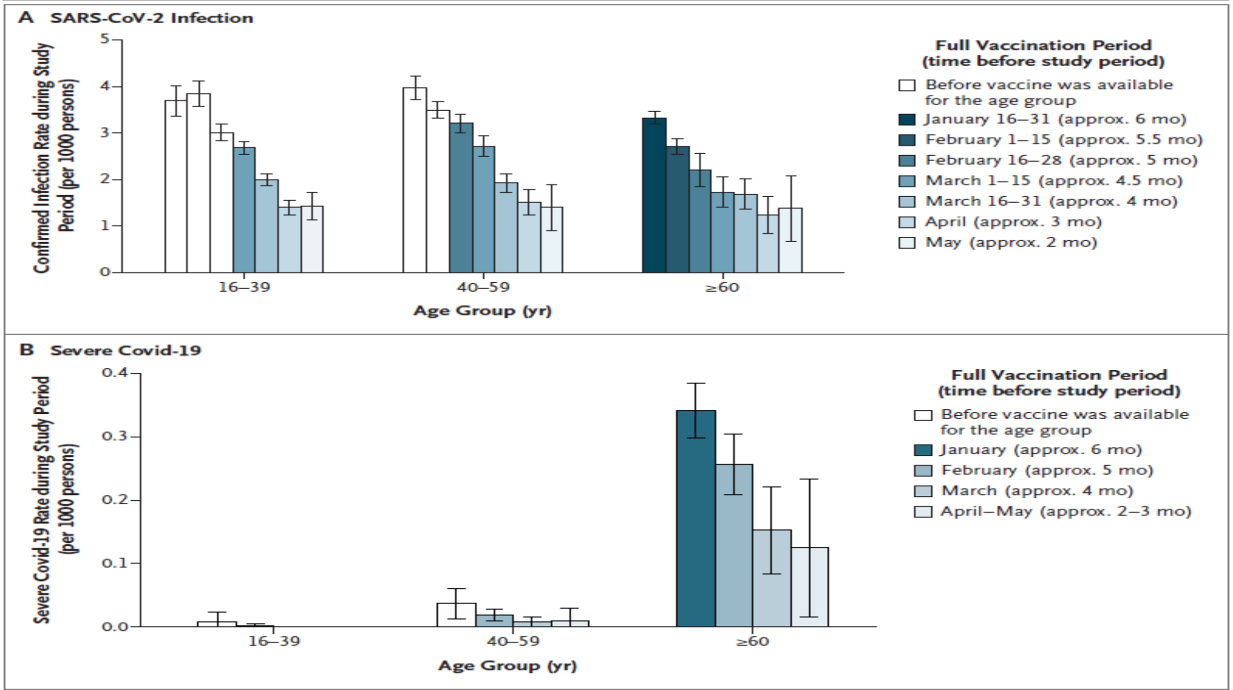

Figure 3. Rates of Documented SARS-CoV-2 Infection and Severe Covid-19, July 11 to 31, 2021.
Shown are the rates of documented infection per 1000 persons (Panel A) and rates of severe Covid-19 per 1000 persons (Panel B), according to period of second dose of Covid-19 vaccine and age group. In the analyses in the age groups younger than 60 years, white bars represent periods during which vaccination was restricted to only designated groups (e.g., health care workers and severely immunocompromised adults). I bars represent 95% confidence intervals, which are not adjusted for multiplicity. In Panel A, white bars represent half a month; in Panel B, white bars represent a month.

Horne et. al (2022) use UK data and report progressive waning by months since second dose for BNT162b2 and ChAdOx1-S, over periods of up to 6 months after second dose, against infection, hospitalization, and death, with BNT162b2 consistently more protective than ChAdOx1-S. This study was excluded because of the non-standard time gap between first and second doses.

Fowlkes et al. (2021) report evidence of waning efficacy over time for healthcare workers at 8 U.S. locations, of whom 65% received Pfizer vaccine, 33% received Moderna, and 2% received J&J. Efficacy against any infection was 85% for 14-199 days after full vaccination; 81% for 120-149 days after full vaccination, and 73% for 150+ days after full vaccination. This study did not satisfy the inclusion criteria because the study does not control for vaccine type and because it studies a specific subpopulation (healthcare workers).

Lin et al. (2021) report population evidence from North Carolina of waning efficacy versus symptomatic infection, and slightly declining efficacy versus hospitalization and death for Pfizer, Moderna, and J&J, with greater durability of protection for Moderna. This study did not satisfy the inclusion criteria because the measured time since the first dose, not since full vaccination.

Risk et al. (2022) report evidence of waning for Pfizer, Moderna, and J&J for both infection and hospitalization, but report data for less than versus more than 6 months since primary vaccination, so their VE levels could not be included in Tables 2 and 3, which use a 120 days cutoff.

6.6. Additional Studies of Waning VE and Booster Effectiveness

We discuss here additional studies reporting evidence on waning VE for initial vaccination and booster effectiveness, which did not meet the inclusion and exclusion criteria for Table 4, sometimes simply because the time periods used could not be mapped onto the periods in Tables 2 and 3.

Sheikh et. al (2021) [49] (Scotland) reports an 83-88% reduction in symptomatic infection risk against the Delta variant after an mRNA booster, relative to vaccinated persons at least 25 weeks after vaccination.

Rosenberg et al. (2021) report evidence of declining efficacy against infection (mostly symptomatic) and less strongly against hospitalization, through August 2021, for BNT162b2, mRNA-1273, and ChAdOx1-S, with stronger declines for BNT162b2, and lower efficacy for ChAdOx1-S. This study reports month of vaccination and several specific weeks of infection, with percentages consistent with those in Tables 2 and 3. It did not meet the inclusion criteria because we could not compute time since vaccination from the data provided.

Starrfelt (2022) report evidence of waning effectiveness against infection for BNT162b2, mRNA-1273, waning effectiveness against hospitalization for BNT162b2 (results for other vaccines not reported due to small sample size) and evidence that VE against hospitalization declines with age (results not separated by vaccine type). We excluded it because of limited, inconsistent reporting by vaccine type.

We excluded Martelluci et. al (2022) due to bizarre reported outcomes that make us suspect severe data collection issues, including a 55% mortality among the hospitalized, and mortality during the pre-Omicron period for persons with a booster dose triple that for persons vaccinated without booster.

Additional References (not cited in the main text)

Baden LR, El Sahly HM, Essink B, et al. Efficacy and safety of the mRNA-1273 SARS-CoV-2 vaccine. *N Engl J Med* 2021; 384: 403–16.

Bajema, KL, Dahl, RM. . . . Thornburg NJ, and Surie D, Comparative Effectiveness and Antibody Responses to Moderna and Pfizer- BioNTech COVID-19 Vaccines among Hospitalized Veterans — Five Veterans Affairs Medical Centers, United States, February 1–September 30, 2021, *Morbidity and Mortality Weekly Report* 2021, 70(49): 1700-1705.

Buchan SA, Seo CY, . . . Yu K, Wilson, SE (2021), Epidemiology of Myocarditis and Pericarditis following mRNA Vaccines in Ontario, Canada: By Vaccine Product, Schedule and Interval, medRxiv working paper, at https://doi.org/10.1101/2021.12.02.21267156;.

Cohn, BA, Cirillo PM, Murphy CC, et al. SARS-CoV-2 Vaccine Protection and Deaths Among US Veterans During 2021, *Science* 2021; DOI: 10.1126/science.abm0620.

Desai, AP, Desai, AP, and Loomis, GJ (2021), Relationship Between Pre-Existing Allergies and Anaphylactic Reactions post mRNA COVID-19 Vaccine Administration, Vaccine 39: 4407-4409.

Folegatti PM, Ewer KJ, Aley PK, et al. Safety and immunogenicity of the ChAdOx1 nCoV-19 vaccine against SARS-CoV-2: a preliminary report of a phase 1/2, single-blind, randomised controlled trial. *Lancet* 2020; 396: 467–78.

Fowlkes A, Gaglani M, Groover K, These MS, Tyner H, Ellingson K (2021), Effectiveness of COVID-19 Vaccines in Preventing SARS-CoV-2 Infection Among Frontline Workers Before and During B.1.617.2 (Delta) Variant Predominance — Eight U.S. Locations, December 2020–August 2021, Morbidity and Mortality Weekly Report 70 (Aug. 24)

Horne, Elsie MF, William J Hulme, Ruth H Keogh, Tom M Palmer, Elizabeth J Williamson, Edward PK Parker, Amelia Green, Venexia Walker, Alex J Walker, Helen Curtis, Louis Fisher, Brian MacKenna, Richard Croker, Lisa

Hopcroft, Robin Y Park, Jon Massey, Jessica Morley, Amir Mehrkar, Sebastian Bacon, David Evans, Peter Inglesby, Caroline E Morton, George Hickman, Simon Davy, Tom Ward, Iain Dillingham, Ben Goldacre, Miguel A Hernan, Jonathan AC Sterne, William J Hulme, Ruth H Keogh, Tom M Palmer, and Elizabeth J Williamson (2022), Waning effectiveness of BNT162b2 and ChAdOx1 COVID-19 vaccines over six months since second dose: a cohort study using linked electronic health records, MedrXiv, https://doi.org/10.1101/2022.03.23.22272804.

Israel A, Merzon E, Schallfer AA, Shenhar Y, Gree I, Golan-Cohen A, Ruppin E,Magen E, Vinker S (2021) Elapsed Time since BNT162b2 Vaccine and Risk of SARS-CoV Infection: Test Negative Study Design, *BMJ*, at BMJ 2021;375:e067873 | doi: 10.1136/bmj-2021-067873 [Leumit Health Clinic]

Martelluci, Cecilia Acuti, Maria Elena Flacco, Graziella Soldato, Giuseppe Di Martino, Roberto Carota, Antonio Caponetti, and Lamberto Manzoli, Effectiveness of COVID-19 Vaccines in the General Population of an Italian Region before and during the Omicron Wave, *Vaccines* 2022, 10, 662, at https://doi.org/10.3390/vaccines10050662.

Merovich D, Anis A, . . ., Keinan-Boke L, Alroy-Preis S (2021), Myocarditis after BNT162b2 mRNA Vaccine Against Covid-19 in Israel, *New England journal of Medicine*, DOI: 10.1056/NEJMoa2109730.

Mizrahi B, Lotan R, Kalkstein N, et al. Correlation of SARS-CoV-2 breakthrough infections to time-from-vaccine; preliminary study. July 31, 2021 (https://www.medrxiv.org/content/10.1101/2021.07.29

Puranik A, Lenehan, PJ, Silvert E, et al. Comparison of two highly-effective mRNA vaccines for COVID-19 during periods of Alpha and Delta variant prevalence. *medRxiv*. 2021 Aug 9;2021.08.06.21261707 (preprint).

Page MJ, McKenzie JE, Bossuyt PM, Boutron I, Hoffmann TC, Mulrow CD, et al. (2020), The PRISMA 2020 statement: an updated guideline for reporting systematic reviews. MetaArXiv. September 14. DOI: 10.31222/osf.io/v7gm2.

Ramasamy MN, Minassian AM, Ewer KJ, et al. Safety and immunogenicity of ChAdOx1 nCoV-19 vaccine administered in a prime-boost regimen in young and old adults (COV002): a single-blind, randomised, controlled, phase 2/3 trial. *Lancet* 2021; 396: 1979–93.

Rethlefsen ML, Kirtley S, Waffenschmidt S, Ayala AP, Moher D, Page MJ, Koffel JB, PRISMA-S Group (2020), PRISMA-S: An Extension to the PRISMA Statement for Reporting Literature Searches in Systematic Reviews, at https://www.equator-network.org/reporting-guidelines/prisma-s/.

Risk, Malcolm Chen Shen, Salim S. Hayek, Lynn Holevinski, Elena Schiopu, Gary Freed, Cem Akin, and Lili Zhao (2022), Comparative Effectiveness of COVID-19 Vaccines against the Delta variant, *Clinical Infectious Disease*, Doi: 10.1093/cid/ciac106.

Rosenberg E, Dorabawila V, Easton D, Bauer UE, Kumar J, oen R, Hoefer D, Wu M, Lutterloh E, Conroy MB, Greene D, Zucker HA (2021), Covid-19 Vaccine Effectiveness in New York State, *N. Engl. J. Med.* at DOI: 10.1056/NEJMoa2116063.

Schultz NH, Sorvoli IH, . . . , Tjonnfjord GE, and Holme PA (2021), Thrombosis and Thrombocytopenia after ChAdOx1 nCoV-19 Vaccination (2021), *New England Journal of Medicine* 384: 2324-30.

Scobie HM, Johnson AG, Suthar AB, et al. Monitoring Incidence of COVID-19 Cases, Hospitalizations, and Deaths, by Vaccination Status — 13 U.S. Jurisdictions, April 4–July 17, 2021. *Morbidity and Mortality Weekly Report*; 70:1-7 (early release Sept. 10, 2021).Starrfelt, Jostein, Anders Skyrud Danielsen, Eirik Alnes Buanes, Lene Kristine Juvet, Trude Marie Lyngstad, Gunnar Øyvind Isaksson Ro, Lamprini Veneti, Sara Viksmoen Watle, and Hinta Meijerink (2022), Age and product dependent vaccineeffectiveness against SARS-CoV-2 infection

and hospitalisation among adults in Norway: a national cohort study, July–November 2021, *BMC Medicine (2022) 20:278*

US Food and Drug Administration (2022, May 5)**,** Coronavirus (COVID-19) Update: FDA Limits Use of Janssen COVID-19 Vaccine to Certain Individuals.

Voysey, Voysey M, Clemens SAC, Madhi SA, et al. Single-dose administration and the influence of the timing of the booster dose on immunogenicity and efficacy of ChAdOx1 nCoV-19 (AZD1222) vaccine: a pooled analysis of four randomised trials. *Lancet* 2021b; 397: 881-91.
